# Adaptive Homeostatic Strategies of Resilient Intrinsic Self-Regulation in Extremes (RISE): A Randomized Controlled Trial of a Novel Behavioral Treatment for Chronic Pain

**DOI:** 10.3389/fpsyg.2021.613341

**Published:** 2021-04-12

**Authors:** Martha Kent, Aram S. Mardian, Morgan Lee Regalado-Hustead, Jenna L. Gress-Smith, Lucia Ciciolla, Jinah L. Kim, Brandon A. Scott

**Affiliations:** ^1^Research Department, Phoenix Veterans Affairs Health Care System, Phoenix, AZ, United States; ^2^Department of Psychology, Arizona State University, Tempe, AZ, United States; ^3^Department of Family, Community, and Preventive Medicine, College of Medicine-Phoenix, University of Arizona, Phoenix, AZ, United States; ^4^Chronic Pain Wellness Center, Phoenix Veterans Affairs Health Care System, Phoenix, AZ, United States; ^5^Department of Educational Psychology, Northern Arizona University, Flagstaff, AZ, United States; ^6^Department of Psychology, Phoenix Veterans Affairs Health Care System, Phoenix, AZ, United States; ^7^Department of Psychology, Oklahoma State University, Stillwater, OK, United States; ^8^Department of Psychology, University of California, San Diego, San Diego, CA, United States; ^9^Department of Psychology, Midwestern University, Glendale, AZ, United States

**Keywords:** homeostasis, chronic pain, self-regulation, resilience, self-growth, intervention, clinical trial

## Abstract

Current treatments for chronic pain have limited benefit. We describe a resilience intervention for individuals with chronic pain which is based on a model of viewing chronic pain as dysregulated homeostasis and which seeks to restore homeostatic self-regulation using strategies exemplified by survivors of extreme environments. The intervention is expected to have broad effects on well-being and positive emotional health, to improve cognitive functions, and to reduce pain symptoms thus helping to transform the suffering of pain into self-growth. A total of 88 Veterans completed the pre-assessment and were randomly assigned to either the treatment intervention (*n* = 38) or control (*n* = 37). Fifty-eight Veterans completed pre- and post-testing (intervention *n* = 31, control = 27). The intervention covered resilience strengths organized into four modules: (1) engagement, (2) social relatedness, (3) transformation of pain and (4) building a good life. A broad set of standardized, well validated measures were used to assess three domains of functioning: health and well-being, symptoms, and cognitive functions. Two-way Analysis of Variance was used to detect group and time differences. Broadly, results indicated significant intervention and time effects across multiple domains: (1) Pain decreased in present severity [*F*_(__1, 56)_ = 5.02, *p* < 0.05, η^2^*_p_* = 0.08], total pain over six domains [*F*_(__1, 56)_ = 14.52, *p* < 0.01, η^2^*_p_* = 0.21], and pain interference [*F*_(__1, 56)_ = 6.82, *p* < 0.05, η^2^*_p_* = 0.11]; (2) Affect improved in pain-related negative affect [*F*_(__1, 56)_ = 7.44, *p* < 0.01, η^2^*_p_* = 0.12], fear [*F*_(__1, 56)_ = 7.70, *p* < 0.01, η^2^*_p_* = 0.12], and distress [*F*_(__1, 56)_ = 10.87, *p* < 0.01, η^2^*_p_* = 0.16]; (3) Well-being increased in pain mobility [*F*_(__1, 56)_ = 5.45, *p* < 0.05, η^2^*_p_* = 0.09], vitality [*F*_(__1, 56)_ = 4.54, *p* < 0.05, η^2^*_p_* = 0.07], and emotional well-being [*F*_(__1, 56)_ = 5.53, *p* < 0.05, η^2^*_p_* = 0.09] Mental health symptoms and the cognitive functioning domain did not reveal significant effects. This resilience intervention based on homeostatic self-regulation and survival strategies of survivors of extreme external environments may provide additional sociopsychobiological tools for treating individuals with chronic pain that may extend beyond treating pain symptoms to improving emotional well-being and self-growth.

**Clinical Trial Registration:** Registered with ClinicalTrials.gov (NCT04693728).

## Introduction

Current treatments for chronic pain provide limited benefit, focus on pathology, and often ignore the resources of the person who experiences chronic pain (Institute of Medicine, 2011; Mardian et al., 2020). Pain is a fundamental biological property of life and can be understood as a part of homeostatic regulation that signals danger to the organism (Cannon, 1932, p. 229). Stability and instability are qualities that characterize chronic pain (Moseley et al., 2003; Rost et al., 2016), as well as predictability of environments (Franch-Gras et al., 2017; Riotte-Lambert and Matthiopoulos, 2019), and homeostatic regulation (McEwen, 1998; Schulkin, 2003; Sterling, 2004). This study will examine chronic pain as a derailed homeostatic process that threatens stability, is aversive and unpredictable, and that shares these characteristics with external extreme environments. Our key questions concern how individuals survive well in deeply aversive, unpredictable environments such as chronic pain; their strategies and characteristics of adaptive survival; and how such strategies can inform this resilience intervention for chronic pain. By situating chronic pain within homeostatic regulation, this study joins the growing interest in interoception and emerging directions in clinical thought and practice (Barrett and Simmons, 2015, p. 419–429; Barrett, 2017, p. 1–23; Tabor et al., 2017, p. 1007–1011, 2020, p. 143–149; Khalsa et al., 2018, p. 501–513; Tabor and Burr, 2019, p. 54–61; Karoly, 2020). This introduction will review: 1. Adaptive survival in extreme environments, 2. Characteristics of adaptive survival, 3. Homeostatic regulation as bivalent process, and 4. An integrated resilient intrinsic self-regulation in extremes (RISE) model for chronic pain.

This study began with our earlier review of adaptive survival as described in narratives of the classical survivor literature of the Gulag, the Holocaust, and prisoner of war (POW) experiences (Kent et al., 2011, p. 591–595, 2015, p. 264–304). In quantitative inquiries of representative autobiographical narratives (*n* = 16), we sought to identify behaviors that helped people persevere under conditions of extreme personal challenges. The survivors’ adaptive skills could potentially inform approaches to chronic mental and physical conditions such as posttraumatic stress disorder (PTSD) and chronic pain.

The samples of survivor experiences originated from very diverse geographic regions, unique historical events, and were separated by decades: the 1930s of the Soviet Union and Stalin’s regime, 1940s and the Holocaust camps in Nazi occupied Poland, and 1960s North Vietnam POW camps at war with the United States (Frank, 1958; Delbo, 1995, p. 142–143; Levi, 1961; Solzhenitsyn, 1963, 1973; Ginzburg, 1981, 2017, p. 220–221; Frankl, 1984, p. 39; Appelbaum, 2003; Public Broadcasting Service, 2010). The survivors’ strategies have three characteristics in common:

1. **Engagement** exemplified by interest, curiosity, appreciation, and noticing beauty.
2. **Social relatedness** captured by expressions of empathy, compassion, helping, friendship, and love.
3. **Self-growth and development** expressed in instances of learning, creativity, ethical, and personal integrity, and self-development in the midst of torture.

These strategies expanded hugely the range of subjective experiences survivors could have beyond the persistent threats and suffering of their captivity. The recall of activities from prior times (e.g., chanting poetry, constructing an imaginary dream house, devising a tapping code for communication) came with positive feelings, independence, agency, and experiences from a vibrant full life that had possibilities.

The environment in captivity can be described as unpredictable, with persistent short-term acutely unpredictable threats and long-term chronic unpredictability that lasted months and years. Predictability and safety were sporadic lulls in the perpetual violence. Prolonged predictability and safety did not exist or existed only in memories of the past in which survivors described stable peaceful homes, stable work roles, and lasting relationships. They imported their past predictable activities and stable environment into captivity. With engagement and relatedness, they created experiences of growth and safety in an environment that was bent on their destruction.

The adaptive activities of the survivors are deeply rooted in homeostasis. In 1865 Claud Bernard noted the existence of an internal environment, the *milieu interièur*, that maintained the “constancy” of internal body fluids and organs in the midst of bustling Parisian life (Gross, 1998, p. 380–385). This concept was codified decades later when the American physiologist Walter Cannon coined the term *homeostasis* and celebrated its feat in *The Wisdom of the Body* (Cannon, 1932). In the examples of their adaptive strategies the survivors responded to their own recalled internal states rather than to the threatening external surroundings. The survivors had found intuitively the feeling states that could establish internal “stability” in the midst of unpredictable surroundings. For nearly a century following work Cannon’s (1932), behavioral and psychological research flourished but homeostatic regulation disappeared or was preempted by the dominant stress research. The past two decades have seen a resurgence of interest in interoception and homeostasis through the introduction of feelings as key elements of homeostatic regulation. Damasio (1994, 2010, p. 48) and A.D. (Bud) Craig, 2002, p. 655–666, 2015) conceived of feelings as representing body states that signaled values of well-being or decline. The aim of internal body feelings was to protect the organism with feelings of positive and negative valence sensing harm or advantage.

Craig described pain as a “homeostatic feeling” (2003, p. 303–307) similar to other body feelings of temperature, hunger, thirst, and itch. He called feelings “interoceptive constructs” (Craig, 2003, p. 303–307) that reflected the cost or benefit values that were bodily registered in homeostasis. Craig articulated the **afferent** homeostatic somatosensory pathway as the missing compliment (body to brain communication–the sensory state of the *body to* the brain) of the extensively studied **efferent** autonomic nervous system (brain to body communication—*brain to* the skeletal motor system for action in the external world). Thus, afferent body feelings informed and shaped actions in the world, a process that started not in the cortex but in body feelings sensed in spinal cord neurons. Craig identified the location of the afferent neurons for heat and pain in lamina I of the anterior spinal cord and the afferent somatosensory tract projecting to brain stem nuclei that had connections with all areas of the body. The upper part of the somatosensory tract ascended as topographic cinemascopic maps and “movie snippets” to the anterior insular cortex (AIC) where feelings and maps became conscious, where a sense of self and agency emerged, and where bivalent processes were organized as positive inclinations that connected with the left hemisphere and negative ones with the right hemisphere. Thus, the parasympathetic processes are represented in the left insula and the sympathetic opponent processes in the right insula. The left insula is activated with energy nourishment, calm behavior, connectedness, prospective responding, and positive affect while the right insula is activated with energy expenditure, challenging behavior, arousal and stress, reactive responding, and negative affect, as summarized in Table 1. The bivalent opponent processes represented in Table 1 served as a basis for understanding the adaptive survival strategies described above from a homeostatic perspective and for development of the therapeutic approach in the RISE intervention. While the efferent aspect of sympathetic branch was already described a century ago by Langley and Cannon and dominated behavioral research on stress, its afferent counterpart is just presently making its appearance in emerging approaches to pain in studies on interoception, Baeysian predictive processes, among others. Recognition of the power of this afferent counterpart led the study team to adapt survival strategies in extremes to harness the power of afferent regulation in the RISE intervention for chronic pain.

**TABLE 1:** Opponent processes of the anterior insular cortex (AIC) and co-activation of anterior cingulate cortex (ACC).

Opponent Processes of the Anterior Insula (AIC) and coactivated Anterior Cingulate	
Left (L) Anterior Insula + Anterior Cingulate	Right (R) Anterior Insula + Anterior Cingulate
Parasympathetic	Sympathetic
Left (L) Anterior Insula + Anterior Cingulate	Electrical stimulation of R produces tachycardia—increased heart rate
Painless distention of stomach activate vagal parasympathetic sensory fibers	Cool stimulation produce sympathetic vasoconstriction
L AIC, ACC, and L amygdala—vagal-activated heart rate variability and oxytocin affiliation and empathy	R AIC, ACC, and R amygdala—sympathetic arousal of fear, stress, cortisol release
L side activation—energy nourishment	R side activation—energy expenditure
L forebrain—positive affect and approach motivation	R forebrain–negative affect and avoidance motivation
L—deactivation of AIC and ACC in depression	R—hyperactivation of AIC and ACC in anger
Calm behavior, energy nourishment	Challenging behavior, energy expenditure
Maternal love, hearing happy voices	Sadness and anticipation of pain
Music and pleasant odors inhibit pain	Pain
Difference in activation for women and men	

The bivalent opponent process is exemplified in the strategies of remarkable adaptive survival. The survivors themselves described their spontaneous methods as making their lives more tolerable in the midst of harsh realities. On closer look, their strategies themselves express features that are not obvious, and for this reason may have been particularly effective in hiding opponent approaches: (1) The *qualities* of the activities are intrinsically rewarding (Fulcher, 2008, p. 11–13; Gottlieb et al., 2013; Gruber et al., 2014, p. 486–496; Leslie, 2014; Glynn et al., 2015, p. 189–202), creative (Scarry, 1999), express curiosity (Berlyne, 1960; Kashdan, 2009; Silvia and Kashdan, 2009, p. 785–797), interest (Silvia, 2005, p. 89–102; Hidi and Renninger, 2006; Greenfield, 2009, p. 111–127), and self-regulation (Renninger and Hidi, 2016; Vittersø, 2016, p. 256), are varied, and promote self-growth (Decety and Ickes, 2009; Cabanac, 2010, p. 113–124; Umberson and Montez, 2010, p. 54–66); (2) The *structure* of survivor activities represent gradients (Frijda, 1986) rather than goal-oriented end points (McNaughton et al., 2016, p. 25–49), show flexibility (Cabanac, 2009), and are simulations of past actions (Hauk et al., 2004, p. 301–307; Keysers et al., 2004, p. 335–346; Decety and Grèzes, 2006, p. 4–14; de Vries and Mulder, 2007, p. 5–13; Svensson et al., 2009, p. 95–114; Ticini et al., 2012, p. 464–474) that are simultaneously scaffolded onto self-regulation strategies (Williams et al., 2009, p. 1257; Clark, 2016; Varga, 2019); *Persons* become agents, actors, “origins” rather than “pawns” in shaping their lives (de Charms, 1968; Nygård and Kunszenti, 1999, p. 91–106; Haggard, 2017, p. 196–207) with coherent narratives that reconstruct experienced stories and harness prospective memory in order to create the future (Schacter et al., 2017, p. 41–50), and that promote self-growth and *eudaimonia* (Waterman, 2013; Vittersø, 2016, p. 253–276; Ryan and Deci, 2017).

Chronic pain is an aversive internal environment that engenders few such adaptive responses and qualities. The effects of aversive unpredictable pain are extensively documented in experimental and clinical paradigms that compare pain-free and clinical populations: unpredictable timing of aversive stimuli produces more anxiety and fear than predictable aversive stimuli (Mineka and Kihlstrom, 1978, p. 256–271; Grillon et al., 2004, p. 916–924); higher variability in the intensity of pain predicts lower quality of life and longer duration of pain (Tupper et al., 2013, p. 563–570); unpredictability combined with individual characteristics of depression, anxiety or intolerance of uncertainty are associated with greater pain (Edwards et al., 2016, p. 70–92; Labrenz et al., 2016, p. 104–114; Meulders and Bennett, 2017, p. 76–87); sleep is often dysregulated and problematic in those with chronic pain, and intertwined with mental health symptoms, such as PTSD, depression, and anxiety (Harding et al., 2018, p. e127-e134; Saconi et al., 2021, p. 101411); unpredictable pain triggers anticipatory pain-related fear and response biases that maintain pain and fear (Schmitz and Grillon, 2012, p. 527–532; Outcalt et al., 2015, p. 535–543; Bélanger et al., 2017, p. 367–372) and generalizes to other contexts in animal studies (Meulders and Bennett, 2017, p. 76–87). For humans, generalization occurs readily in contexts that are perceptually or conceptually similar to the original pain experience (Dunsmoor and Murphy, 2015).

Two main parameters emerge from the survivor and pain literatures that define the environment as unpredictable or predictable and responses as controlled intrinsically or extrinsically. The interaction of these parameters results in a four-way pairing of environment and biobehavioral control that brings to the fore: (1) the persistence of pathology in reactive responses that cannot be stopped easily despite safety (as in PTSD) or absence of tissue damage (as in chronic pain) and (2) the presence of resilient adaptive regulation where prospective responses can stop and inhibit the reactivity of threat and pain. The resilient adaptive responses to threat identified in the survivor strategies open the door to the possibility of training engagement and relatedness responding as opponent processes to modulate the reactivity of pain or anxiety.

**FIGURE 1:**
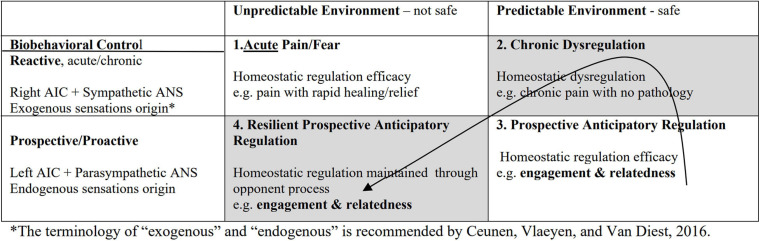
Environment × Biobehavioral Control in Interaction.

To reproduce the experience of adaptive regulation in an uncertain environment, our earlier study on PTSD (Kent et al., 2011, p. 591–595, 2015, p. 264–304) asked participants to re-experience engagement or relatedness episodes from their past safe environment, as represented by **Quadrant 3**, then take these experiences into trauma of **Quadrant 2**, and transform reactivity into resilient action represented in **Quadrant 4**, thereby emulating the adaptive survivor strategies in a route shown by the arrow in Figure 1.

A century of research findings support the first three quadrants. **Quadrant 1** is represented by work on sympathetic models of acute pain and stress (Pawlik, 1997, p. 91–96; Labrenz et al., 2016, p. 104–114), **Quadrant 2** by psychobiology and dysregulation in psychopathology (Sharp and Harvey, 2001, p. 857–877; Asmundson et al., 2002, p. 930–937; Jovanovic et al., 2010, p. 244–251; de Leon, 2014, p. 492–494), and **Quadrant 3** by animal and human learning (Christianson et al., 2011, p. 458–464; Maier and Seligman, 2016, p. 349–367; Schacter et al., 2017, p. 41–50). Resilience of **Quadrant 4** is the most recent area of inquiry (Rutter, 1979, p. 49–74; Masten and O’Connor, 1989, p. 274–278; Luthar and Cicchetti, 2000, p. 857–885; Sturgeon and Zautra, 2010, p. 105–112; Tops et al., 2014, p. 31–38). Though novel, our model draws from extensive historical data combined with current theories of pain.

The purpose of the present study is to test the RISE therapeutic intervention in patients with chronic pain. This therapeutic intervention is based on the model of self-regulation illustrated in Figure 1 which captures the spontaneous survivor strategies in the context of extreme threatening environments. Survivors drew on earlier peace-time experiences to help them endure insurmountable threats. These resources are inherent endowments of homeostasis which is the dynamic intrinsic force that maintains resilience as the organism interacts with the challenges of its environment. The intervention methods of this study emulate the characteristics of adaptive survival in a step-wise process in which participants will identify engagement, social relatedness, and self-growth from earlier times of their lives, practice these in the context of pain, and thereby transform pain into growth and a future good life.

Existing psychological therapies for pain including Cognitive Behavior Therapy (CBT), Acceptance and Commitment Therapy (ACT), and therapies that focus on fear avoidance and motivation are directed at modifying pain symptoms and changing the pain/body/environment. This leaves a major gap in current treatments for pain in that there is an absence of life growth approaches that treat pain as an opportunity for personal growth. The RISE intervention fills this gap by offering a treatment that aims to change the manner of living in one’s environment; it changes the person, the feelings, interests, and relatedness with which the person lives.

To test the efficacy of this intervention, the outcome measures included well-validated tests to assess pain symptoms, mental health symptoms, well-being, and cognitive functions. Within the broad area of pain subareas of pain intensity, mobility, vitality, and catastrophizing were measured as possible intervention effects. Mental health domains included depression, anxiety, and post-traumatic stress symptoms. Given the wide-ranging effects of chronic pain, insomnia and overall somatic symptoms would also be tested. Well-being was assessed to capture a possible resilience response to RISE. Cognitive functions were covered by relevant subtests of larger neuropsychological batteries that include domains of new learning, executive functioning, and working memory.

The test of this intervention is expected to have broad effects on well-being and positive emotional health, to improve cognitive functions, and to reduce pain symptoms in comparison to a waitlist control condition and thus help to transform the suffering of pain into self-growth. We predict: (1) gains in well-being and self-growth, (2) decreases in symptoms of dysregulation such as in pain, depression, and anxiety, and (3) improved cognitive functions such as working memory or executive functions.

## Methods

### Intervention Development

Stability and its maintenance is the common focus of adaptive survival and of homeostasis. Research activities over the past two decade have identified the mechanisms of behavioral homeostatic regulation: in homeostatic feelings of valence that allow conscious re-experiencing of feelings from different times, in topographic cinemascopic narratives, and in bivalent processes that render conscious and visible the opponent processes that maintain stability. Survivor narratives demonstrate the flexibility of homeostatic feelings that allow sampling from different times of people’s lives, different contexts from distant places, and different value systems.

In the spontaneous examples of adaptive survival we could recognize the striving to maintain balance. We have followed these homeostatic tracts in developing this intervention. The gradient qualities of the survivor examples have allowed our approach to be more open-ended and individually tailored to the person’s unique inclinations, response tendencies, and lived contexts. It allows openness where participants can modify and develop new emotional, cognitive, and behavioral structures during the course of their participation.

### Program Materials

The implementation strategy followed an intuitive flow of emotional restoration and growth by starting with a preparatory step. To make room for the practice of engagement and relatedness at the outset, participants were asked to set aside pain and suffering; these would be addressed later after a period of emotional rebuilding and re-experiencing safety from the past. Instead, participants were asked to find one example in which they were valued, cherished, and loved or they cherished and loved someone or something else. They were to describe the example in detail along with their sensory impressions. They were to turn to this episode whenever they felt pain or distress rather than remaining in their pain or distress and have these interfere with the emotions of engagement and relatedness. After this step, the full program began with engagement and relatedness, followed by applying these in transformations, and concluding with the making of a good life.

The intervention process and content were manualized and organized into four modules. The first two modules focused on rebuilding engagement and social relatedness with the re-experiencing of childhood and early adulthood examples and making connections with current engagement and relatedness. Module III turned to the pain that was set aside earlier. In this module participants were asked to take engagement and social relatedness into the unpredictable environment of chronic pain and related trauma where they were asked to focus on engagement or relatedness and thereby transform suffering into growth. Module IV asked participants to consider the qualities of a good life, design a good life, and apply the new learning to their own lives. The program was delivered over 8 weeks of training; each module was implemented in 2 weekly segments, during 90 min sessions.

**Module I** asks participants to provide examples of engagement, defined as interest, curiosity, appreciation, and noticing beauty. They are to describe each example in detail and indicate what their five senses were doing. They are also asked to describe the actions on a printed theater stage. As a last activity, participants are to make a visual representation of engagement in a modality of their choosing, such as a collage, family album, a painting, etc.

**Module II** covers social relatedness defined as empathy, compassion, helping, friendship, and love. Similar to Module I, participants are asked to find examples of relatedness, describe these, indicate what their five senses were doing, and imagine them on a stage. Again, a visual representation is encouraged.

**Module III** focuses on applying and taking engagement and social relatedness into experiences of pain and transforming pain into approach and engagement responses. Participants are asked to make a list of their pain environment barriers (physical, emotional, or social/cultural). For each barrier they are to discuss a resource from engagement and social relatedness that can be used to overcome the barrier. This format is used to integrate the new strategies with participants’ own strategies that have worked well and others that need improvement with the intent to expand approaches to living more fully despite pain.

**Module IV** covers building a good life with the new resources. Participants are to list present pain environments (physical, emotional, social/cultural). Next to each they are to discuss the resources available to them or that they could establish. They are to consider barriers and explore possibilities for reinventing themselves beyond pain and to conclude the program by writing their own reinvention story.

Handouts were developed for each module. Module I and Module II include prepared forms in which participants describe their examples of engagement, social relatedness, self-growth and the reactions of their five senses. Additional handouts incorporate literature excerpts which provided examples of engagement, relatedness, and self-growth.

The exercise, called “Life on Stage,” asks participants to place their examples of engagement or relatedness on stage and see themselves as re-enacting the episode of engagement or social relatedness. The stage background remains that of the examples. The “Life on stage” exercise consists of several stages that represent: inner stage of mind and body experiences, the interpersonal stage of interactions, and the larger group/culture stage and its institutions. Life on Stage captures particularly well the multidimensional sociopsychobiological layers of people’s lives, starting from private mind/body experiences and expanding to the increasingly larger spheres of group and cultural institutions.

In Module III, participants now return to their pain and related traumas and identify three kinds of pain: body, interpersonal, and group/cultural. They return to these not with their old responses but with the engagement or social relatedness episodes they have practiced. The physical pain itself now becomes more distant, remote, and not as immediately present. (e.g., Veteran returns to pain episode from a scene when he was 5 years old and held his lost dog. The background at age five is now in the foreground and the pain experience is more remote). Participants complete this activity in provided handout forms.

Module IV asks participants to design a good life. The resources and skills now become part of daily life and the future while pain is in the background. The Greek definition of a good life consists of vital abilities exercised to levels of excellence in a life that has scope or opportunities (Hamilton, 1973, p. 22–37). However, pain has no opportunities, life is diminished, and opportunities must be created. Designing a good life in this exercise is achieved with engagement, relatedness, and additional resources uncovered during the program. With the new resources, participants develop plans for present and future pain/trauma barriers in various contexts and create their own resilient life story.

### Participants

To allow for broad Veteran participation, eligibility criteria covered a wide age range of 18–80 and military conflicts covering Vietnam to Operation Enduring Freedom (OEF) and Operation Iraqi Freedom (OIF). Exclusion criteria focused on cognitive and emotional capacities to participate fully in the intervention. Appropriate screens were included for suicidality (Posner et al., 2011, p. 1266–1277), alcohol use (Bradley et al., 2007, p. 1208–1217), and psychosis (Degenhardt et al., 2005). Also excluded were current disabling illness, neurocognitive conditions, inability for small group participation, and concurrent psychotherapies. Attendance requirements allowed 2 missed sessions out of 8. Limited individual makeup sessions were available.

Eligibility criteria included: 18–80 years of age, United States Veterans from the conflicts of OEF/OIF to Vietnam era, and either self-identification as having chronic pain or have one or more chart diagnoses of chronic pain. Exclusion criteria covered: active suicidality (of suicidal intent requiring a greater than outpatient level of care, assessed with the Columbia Suicidality Rating Scale screen, C-SSRS), active alcohol abuse (assessed with Alcohol Use Disorder Identification Test, AUDIT), active psychosis (assessed with Psychosis Screener), current severe disabling illness, inability to participate in a small group interactive setting, inability to meet attendance requirement, neurocognitive conditions other than TBI (e.g., dementia, Parkinsons, stroke), and currently receiving Exposure Therapy, Cognitive Behavior Therapy, or Acceptance and Commitment Therapy.

A total of 88 participants provided written consent and were assessed for eligibility. Of these 13 were lost to screen failures. The remaining 75 participants completed the pre-assessment and were randomly assigned and stratified by sex: male *n* = 52, female *n* = 23; intervention *n* = 38, control *n* = 37.

A total of 58 participants completed pre- and post-testing and the entire intervention: Intervention *n* = 31, control = 27. The remaining 17 participants were lost to follow-up or dropped out for a variety of reasons related to work demands, scheduling conflicts, or Arizona’s summer heat.

Sample characteristics of the 75 participants who passed eligibility screening were representative of the population of Veterans seeking medical care at the Carl T. Hayden Medical Center. Age of participants ranged from 27 to 73 (*M* = 56.98). Women Veterans were somewhat disproportionately represented in participation in this research study. However, this is also a typical finding in that women Veterans tend to participate in group interventions at higher rates relative to male Veterans. This sample of Veterans also endorsed a relatively high level of education. Sample characteristics are described in Table 2.

**TABLE 2:** Sample characteristics of Veterans participating in this study.

	Intervention (*n* = 0 43)			Control (*n* = 0 32)		

Characteristic	M (SD)	n	%	M (SD)	n	%
Age	58.37 (9.64)			54.59 (11.17)		
**Gender**						
Male		27	62.8		23	71.9
Female		16	37.2		9	28.1
**Education (years)**	15 (1.84)			15 (1.59)		
High school		2	4.7		2	6.3
Some college/AA		22	51.2		16	49.9
College degree		11	25.5		11	34.4
Advanced degree		8	18.6		3	9.4
**Ethnicity**						
White/non-Hispanic		37	86		23	71.9
Hispanic		14	14		6	18.8
Did not respond		–	–		3	9.4
**Race**						
White		29	67.4		28	87.5
Black/African American		6	14		4	12.5
Hispanic		6	14		–	–
Asian		1	2.3		–	–
Did not respond		1	2.3		–	–

### Procedures

Study procedures were approved by the institutional review board of the Phoenix VA Health Care System and registered with ClinicalTrials.gov (NCT04693728). Individuals were recruited by clinic announcements and flyers posted at the Carl T Hayden VA Medical Center. Potential participants were consented, then screened for eligibility, and pretested, after which they were randomized to intervention or control conditions. Since this was a group intervention, 20 participants (a range of 10–24) were randomized at the same time for each successive wave. Pre- and post-assessments included completion of standardized questionnaires that assessed positive mental health, mental health symptoms, and pain. Neuropsychological tests assessed working memory, episodic memory, complex attention, and executive functions of inhibition, cognitive flexibility, and speed. Participants in both conditions within a study wave completed post-assessment questionnaires and neuropsychological assessment within a window of 2 weeks after the final treatment or waitlist conditions. The participants in the waitlist conditions received the same intervention training after their waiting period and post testing, however, subsequent post-testing following their intervention training was not completed.

The intervention was implemented in four Waves. Wave I had 8 intervention participants and 7 controls; Wave II had 7 intervention participants and 7 controls; Wave III had 7 intervention participants and 7 controls; Wave IV had 9 intervention participants and 6 controls. Average attendance for the combined Waves was 7.4 sessions out of 8 weekly sessions. The high retention and weekly attendance rate were supported by individual makeup sessions. All group meetings and individual testing sessions were conducted at the Carl T Hayden VA Medical Center.

**FIGURE 2:**
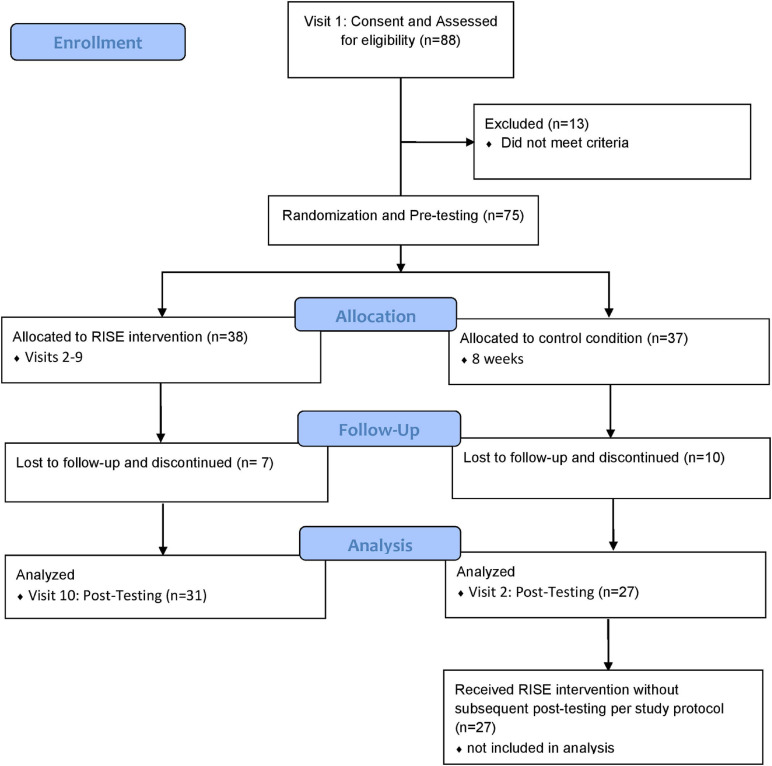
CONSORT Flow Diagram.

The implementation of the intervention is summarized in Figure 2 in the flow of activities that started with enrollment, consenting, screening for eligibility, randomization into intervention and wait list controls in four consecutive Waves (each Wave the *n* = 10–24), followed by pre-testing, 8 week intervention, followed by post-testing. Pre-testing and post-testing were limited to a window of 2 weeks prior to start of training and 2 weeks following training.

### Measures

The efficacy of the intervention was assessed with standardized, well validated measures that were chosen to assess three domains of functioning: level of health and well-being, symptom measures, and cognitive functions that appeared affected by chronic pain.

#### Well-Being Measures

Pre- to post-changes in positive emotional health was assessed with subscales of Mental Health and Physical Health of the RAND 36-item Health Survey (SF-36) (Ware et al., 1994). The Well-Being subscale of the SF-36 mainly assesses a range of affects experienced during the past 4 weeks. The content of the items represent a mix of positive and negative items from feeling nervous and down to calm and happy. These items are rated from 0 (scored as zero) representing highest mood quantity (all the time) to 6 (scored as 100) representing lowest mood quantity (none of the time). The negative and positive items with response ranged 0–6 are weighted in increments of 20 and are thus counterbalanced. Internal consistency was acceptable (α = 0.90).

#### Symptom Measures

Symptoms were assessed with PTSD Check List-5 (PCL-5, α = 0.94) (Weathers et al., 2013), Patient Health Questionnaire, Depression Scale (PHQ-9, α = 0.87) (Kroenke et al., 2001, p. 606–613), Generalized Anxiety Disorder (GAD-7, α = 0.90) (Sptizer et al., 2006, p. 1092–1097), Pain Catastrophizing Scale (PCS, α = 0.92) (Sullivan et al., 1995, p. 525–532), Physical Symptoms Scale (PHQ-15, α = 0.77) (Kroenke et al., 2002, p. 258–266), Insomnia Severity Index (ISI, α = 0.93) (Bastien et al., 2001, p. 297–307), West Haven-Yale Multidimensional Pain Inventory (WYMPI, α = 0.80) (Kerns et al., 1985), Pain Outcome Questionnaire (POQ, α = 0.78) (Clark et al., 2003, p. 381–396). Reliability for the entire symptom measures were in the acceptable range.

#### Neuropsychological Tests

Executive functions assessed with the Word Generation subtest of the Neuropsychological Assessment Battery (NAB) (Stern and White, 2003), the Category Fluency, Category Switching, and Color-Word Switching subtests of the Delis-Kaplan Executive Function System (D-KEFS) (Homack et al., 2005, p. 599–609), Repeatable Battery for the Assessment of Neuropsychological Status (RBANS) (Randolph et al., 1998, p. 310–319) subtests assessed working memory, episodic memory, and complex attention. Alternative versions of all cognitive tests were employed at pre- and post-assessments.

### Statistical Methods

Preliminary analyses, using *t*-tests, examined sample characteristics and baselines scores of clinical outcome variables. *Post hoc* power analyses were also completed in GPower. Primary analyses utilized two-way Analysis of Variance (ANOVA) to examine the effect of treatment group (treatment, no treatment) and time (pre-treatment, post-treatment) on clinical outcomes, including aspects of pain, emotional well-being, psychological symptoms, and neuropsychological functioning. Significant interaction effects were followed by tests of simple main effects. Partial eta-squared (η^2^*_p_*) were used to interpret effect sizes for two-way ANOVAs (small = 0.01, medium = 0.06, large = 0.14. A *post hoc* power analysis was conducted in GPower (Erdfelder et al., 1996). Using assumptions of power (1–β) set at 0.80, α set at 0.05, two-tailed, and a large effect size (within-group, pre-test/post-test mean comparison in ANOVA, partial eta squared set at 0.05), an *n* of approximately 40 would be needed to obtain the recommended statistical power to observe such an effect (Cohen, 1988).

## Results

Preliminary results found no significant group baseline differences between intervention and control subjects in gender, age, education, race, or ethnicity. Further, *t-*tests revealed no significant differences on baseline scores across all clinical outcome measures. The results for the ANOVAs are grouped into four sets of findings:

1. Pain intensity, present moment: Pain Severity (WHYMPI).
2. Pain comprehensive totals: Total Pain (POQ), and Total Pain Decrease (SF-36).
3. Pain negative affect: Pain Negative Affect (POQ), Pain Fear (POQ), and Pain Affective Distress (WHYMPI).
4. Pain mobility, vitality, and wellbeing: Pain Mobility (POQ), Pain Vitality/Energy (POQ), and Emotional Well-being (SF-36).

### Pain Intensity

#### WHYMPI Pain Severity

ANOVA results examining WHYPMI Pain Severity indicated a significant [Group × Time] interaction [*F*_(__1, 56)_ = 5.02, *p* < 0.05, η^2^*_p_* = 0.08]. Simple slope analyses indicated a significant simple main effect of time for the treatment group [*F*_(__1, 56)_ = 6.27, *p* < 0.05, η^2^*_p_* = 0.10], but not for the non-treatment group [*F*_(__1, 56)_ = 0.53, *n.s.*, η^2^*_p_* = 0.01], indicating that WHYPMI Pain Severity decreased significantly for the treatment group from pre-treatment to post-treatment, whereas non-significant positive change was found for the non-treatment group. A simple main group effect was found at post-treatment only [*F*_(__1, 56)_ = 5.11, *p* < 0.05, η^2^*_p_* = 0.07], with the treatment group having significantly lower WHYPMI Pain Severity scores.

### Pain Comprehensive Totals

#### Total Pain POQ

ANOVA results examining Total Pain POQ scores indicated a significant [Group × Time] interaction [*F*_(__1, 56)_ = 14.52, *p* < 0.01, η^2^*_p_* = 0.21], as well as a significant main effect of time [*F*_(__1, 56)_ = 6.02, *p* < 0.05, η^2^*_p_* = 0.10]. Simple slope analyses indicated a significant simple main effect of time for the treatment group [*F*_(__1, 56)_ = 21.08, *p* < 0.01, η^2^*_p_* = 0.27], but not for the non-treatment group [*F*_(__1, 56)_ = 0.86, *n.s.*, η^2^*_p_* = 0.01], indicating that POQ total decreased significantly for the treatment group from pre-treatment (M = 87.71) to post-treatment (M = 70.77), whereas no significant change was found for the non-treatment group. A simple main group effect was found at post-treatment only [*F*_(__1, 56)_ = 5.93, *p* < 0.05, η^2^*_p_* = 0.10], with the treatment group having significantly lower POQ total scores.

#### Total Pain Decrease SF-36

ANOVA results examining SF-36 Pain Decrease indicated a significant [Group × Time] interaction, [*F*_(__1, 56)_ = 6.82, *p* < 0.05, η^2^*_p_* = 0.11]. Simple slope analyses indicated a significant simple main effect of time for the treatment group [*F*_(__1, 56)_ = 11.4, *p* < 0.01, η^2^*_p_* = 0.17], but not for the non-treatment group *[F*_(__1, 56)_ = 0.18, *p* < 0.01, η^2^*_p_* = 0.00], indicating that SF-36 Pain decreased significantly for the treatment group from pre-treatment to post-treatment, whereas little change was found for the non-treatment group. A simple main group effect was found at post-treatment only [*F*_(__1, 56)_ = 7.05, *p* < 0.05, η^2^*_p_* = 0.11], with the treatment group having significantly higher SF Pain Decrease scores.

**FIGURE 3:**
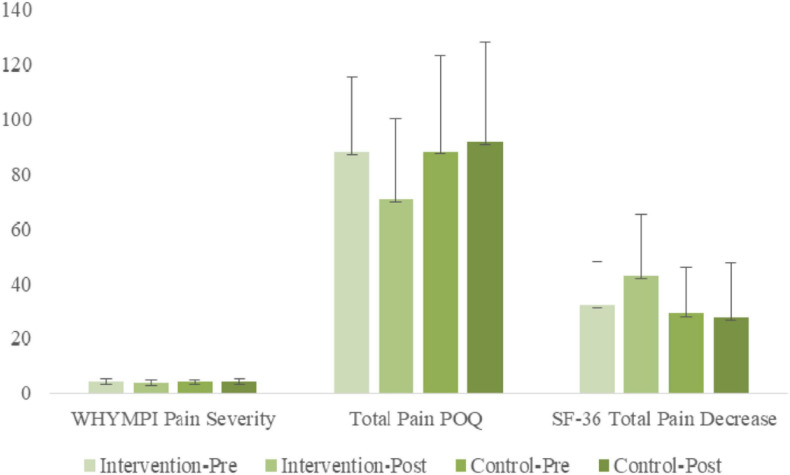
Pre- and post-treatment comparisons of treatment and control groups on WHYMPI Pain Severity-momentary, Total Pain POQ, and SF-36 Total Pain Decrease.

The bar graphs of Figure 3 capture the comprehensive improvements in the chronic pain measures.

### Pain Negative Affect

#### POQ Negative Affect

ANOVA results examining POQ Vitality indicated a significant [Group × Time] interaction [*F*_(__1, 56)_ = 7.44, *p* < 0.01, η^2^*_p_* = 0.12]. Simple slope analyses indicated a significant simple main effect of time for the treatment group [*F*_(__1, 56)_ = 7.43, *p* < 0.01, η^2^*_p_* = 0.12], but not for the non-treatment group [*F*_(__1, 56)_ = 1.41, *n.s.*, η^2^*_p_* = 0.02], indicating that POQ Negative Affects decreased significantly for the treatment group from pre-treatment to post-treatment, whereas little change was found for the non-treatment group. A simple main group effect was found at post-treatment only [*F*_(__1, 56)_ = 4.67, *p* < 0.05, η^2^*_p_* = 0.08], with the treatment group having significantly lower POQ Negative Affect scores.

#### POQ Fear

ANOVA results examining POQ Fear indicated a significant [Group × Time] interaction [*F*_(__1, 56)_ = 7.70, *p* < 0.01, η^2^*_p_* = 0.12]. Simple slope analyses indicated a significant simple main effect of time for the treatment group [*F*_(__1, 56)_ = 10.16, *p* < 0.01, η^2^*_p_* = 0.15], but not for the non-treatment group [*F*_(__1, 56)_ = 0.67, *n.s.*, η^2^*_p_* = 0.01], indicating that POQ Fear decreased significantly for the treatment group from pre-treatment to post-treatment, whereas little change was found for the non-treatment group. Simple main group effects were not significant at either time point.

#### WHYPMI Affective Distress

ANOVA results examining WHYPMI Affective Distress indicated a significant [Group × Time] interaction [*F*_(__1, 56)_ = 10.87, *p* < 0.01, η^2^*_p_* = 0.16]. Simple slope analyses indicated a significant simple main effect of time for the treatment group [*F*_(__1, 56)_ = 13.84, *p* < 0.01, η^2^*_p_* = 0.20], but not for the non-treatment group [*F*_(__1, 56)_ = 1.08, *n.s.*, η^2^*_p_* = 0.02], indicating that WHYPMI Affective Distress decreased significantly for the treatment group from pre-treatment to post-treatment, whereas little change was found for the non-treatment group. A simple main group effect was found at post-treatment only [*F*_(__1, 56)_ = 8.63, *p* < 0.01, η^2^*_p_* = 0.13], with the treatment group having significantly lower WHYPMI Affective Distress scores.

**FIGURE 4:**
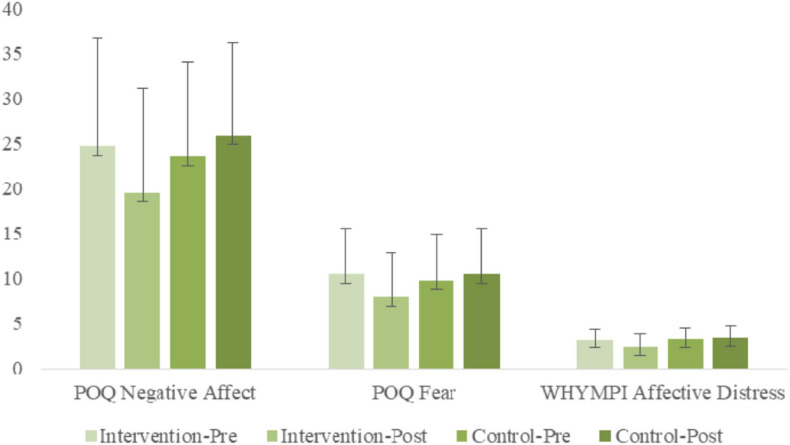
Pre- and post-treatment comparisons of treatment and control groups on negative affect variables: POQ Negative Affect; POQ Fear; WHYMPI Affective Distress.

An overview of the several negative affect measures is captures in the bar graphs of Figure 4.

### Pain Mobility, Vitality, and Well-Being

#### POQ Mobility Interference

ANOVA results examining POQ Mobility indicated a significant [Group × Time] interaction [*F*_(__1, 56)_ = 5.45, *p* < 0.05, η^2^*_p_* = 0.09]. Simple slope analyses indicated a significant simple main effect of time for the treatment group [*F*_(__1, 56)_ = 9.25, *p* < 0.01, η^2^*_p_* = 0.14], but not for the non-treatment group [*F*_(__1, 56)_ = 0.72, *n.s.*, η^2^*_p_* = 0.00], indicating that POQ mobility decreased significantly for the treatment group from pre-treatment to post-treatment, whereas little change was found for the non-treatment group. Simple main group effects were not significant at either time point.

#### POQ Vitality Impairment

ANOVA results examining POQ Vitality indicated a significant [Group × Time] interaction [*F*_(__1, 56)_ = 4.54, *p* < 0.05, η^2^*_p_* = 0.07]. Simple slope analyses indicated a significant simple main effect of time for the treatment group [*F*_(__1, 56)_ = 4.71, *p* < 0.05, η^2^*_p_* = 0.08], but not for the non-treatment group [*F*_(__1, 56)_ = 0.79, *n.s.*, η^2^*_p_* = 0.01], indicating that POQ Vitality decreased significantly for the treatment group from pre-treatment to post-treatment, whereas little change was found for the non-treatment group. Simple main group effects were not significant at either time point.

### Well-Being

#### SF-36 Emotional Well-Being (EWB)

ANOVA results examining SF Emotional Well-Being (EWB) indicated a significant [Group × Time] interaction [*F*_(__1, 56)_ = 5.53, *p* < 0.05, η^2^*_p_* = 0.09]. Simple slope analyses indicated a significant simple main effect of time for the treatment group [*F*_(__1, 56)_ = 4.01, *p* = 0.05, η^2^*_p_* = 0.07], but not for the non-treatment group [*F*_(__1, 56)_ = 1.81, *n.s.*, η^2^*_p_* = 0.03], indicating that SF EWB increased significantly for the treatment group from pre-treatment to post-treatment, whereas a non-significant decrease was found for the non-treatment group. A simple main group effect was found at post-treatment only [*F*_(__1, 56)_ = 4.12, *p* < 0.05, η^2^*_p_* = 0.07], with the treatment group having significantly higher SF EWB scores.

**FIGURE 5:**
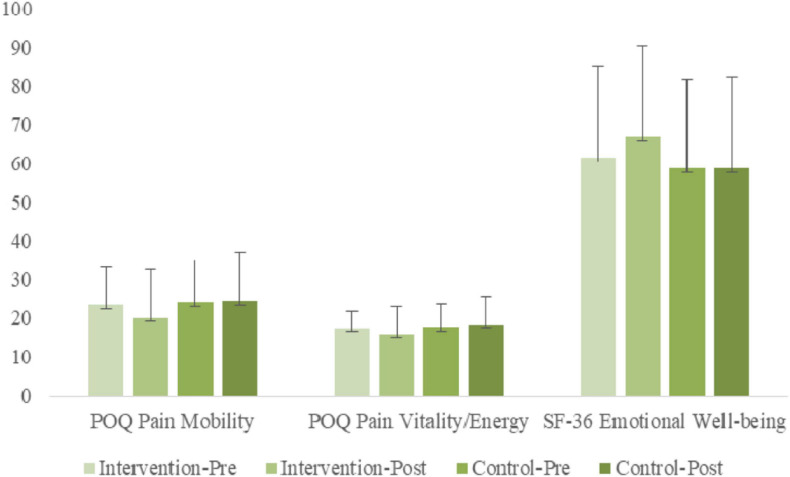
Pre- and post-treatment comparisons of treatment and control groups on strengths variables: POQ Pain Mobility is Pain Impairment in Mobility; POQ Pain Vitality/Energy is Pain Impairment in Vitality/Energy; Emotional Well-being of Positive Emotions.

An overview of the gains in mobility, vitality, and emotional well-being are shown in the bar graphs of Figure 5.

Additional data were collected on the nature and quality of chronic pain experienced by a subsample of 20 participants from the study sample. An analysis of this subsample indicated that pain was highly widespread and chronic. The average duration of pain was 17 years and these subjects experienced pain in an average of 5 different body locations, with pain attributed to numerous illnesses and chronic conditions (range 1–8 pain conditions). The most frequent pain conditions included osteoarthritis, degenerative disc disease, spinal stenosis, fibromyalgia, and neuropathy.

The above results were obtained with the two comprehensive multi-dimensional pain scales, notably the POQ and the WHYMPI, along with the multi-dimensional Health and Wellness SF-36 scale. Overall means did reveal that Veterans presented with moderate insomnia, PTSD, and depressive symptoms. No significant group findings emerged from the short clinical mental health symptom measures of anxiety, PTSD, depression, somatic symptoms, and insomnia. Group findings were also not significant across the cognitive measures assessing executive functions, working memory, and new learning. The obtained results demonstrate robust confirmation of the health and wellness hypothesis supported by a number of related measures detailed above, robust confirmation of reduced symptoms of pain by immediate, overall, and total pain measures, no support for reduced clinical symptoms of anxiety, PTSD, depression, somatic symptoms and insomnia, and no support for improved neuropsychological functions. A summary of overall results is provided in Table 3.

**TABLE 3:** Means, standard deviations, and ANOVA results for intervention and control groups.

	Pretreatment				Posttreatment				ANOVA		
	Intervention (*n* = 31)		Control (*n* = 27)		Intervention (*n* = 31)		Control (*n* = 27)		*F* group	*F* time	*F* [group × time]
**Measures**	***M***	***SD***	***M***	***SD***	***M***	***SD***	***M***	***SD***			
Pain severity (WHYPI_PSev)	4.16	1.08	4.23	0.80	3.76	1.21	4.36	0.91	1.91	1.38	5.03*
Pain affective distress (WHYP_AD)	3.23	1.40	3.27	1.26	2.48	1.35	3.49	1.25	2.79	3.17	10.87*
Pain (POQ) total	87.71	27.50	88.11	35.13	70.77	29.23	91.78	36.43	1.80	6.03*	14.53***
Pain interference in mobility (POQ-MOB)	23.71	9.50	24.22	10.80	20.16	11.64	24.67	12.55	0.80	3.30	5.45*
Pain interference in vitality (POQ-VIT)	17.48	4.34	17.70	6.15	15.97	5.63	18.37	7.30	0.80	0.69	4.54*
Pain negative affect (POQ-NA)	24.74	12.08	23.48	10.55	19.58	11.62	25.89	10.43	0.93	0.99	7.44**
Pain Fear (POQ-F)	10.45	5.11	9.81	5.09	7.90	4.96	10.52	5.06	0.69	2.48	7.71*
Total pain decrease (SF-36 Pain)	32.18	16.08	29.17	16.67	42.58	22.39	27.78	19.69	4.02*	3.99	6.82*
Emotional well-being (SF-36)	61.42	23.52	58.67	22.87	66.97	22.39	54.67	23.72	1.72	0.15	5.53*

*p < 0.05, **p < 0.01, ***p < 0.001.

## Discussion

We propose RISE as a suitable therapeutic opponent for chronic pain; one that is resilient as part of the opponent process of homeostatic regulation (Craig, 2015), that is intrinsic in originating from within the person rather than from external sources, and that follows gradients rather than aimed at end states. RISE differs from self-regulation for achieving extrinsic goals through willpower proposed by Baumeister and Alquist (2009, p. 21–33) and Baumeister and Tierney (2011) or the regulation of emotions through common sense strategies of Gross (2014) that involve the activation of a goal and changing its emotional trajectory at five points: the trajectory, the situation, attention, appraisal, and the response. Our focus is on RISE in response to aversive unpredictable environments, be these internal or external. In this approach, engagement and social relatedness are powerful resources because they express an open, welcoming, independent agency in the form of a flexible gradient suitable for exploration and information gathering in ambiguous environments. Such a stance is internally generated and intrinsically rewarding rather than made in response to extrinsic rewards. This orientation welcomes an exploratory approach to self-regulation, and faces challenges with equanimity, interest or curiosity rather than feeling overwhelmed, helpless, and lacking in resources. The body’s mechanism that allows engagement and relatedness to function in this resilient manner is the opponent process of homeostatic regulation (Craig, 2015), a process that is efficacious in decreasing pain and related limitations.

Although too huge for a comprehensive review, the body of pain research can be organized around several topic areas, is summarized Table 4 and covers: the biomedical approach, brain imaging, felt experience of pain, reactions to pain, individual differences of traits and mental health, social context of pain, therapeutic approaches, and proactive agentic approaches to pain.

**TABLE 4:** Overview of main content areas covering research approaches in the study of pain.

Research domains for investigations and interventions of chronic pain	
Biomedical model	Structural pathology paradigm, pain as nociception
Brain imaging	Localized functions, neural networks, brain plasticity
Felt experience of pain	Intensity of pain, specificity, unpleasantness, predictability; assessments by rating scales
Reactions to pain–*reactive*	Learning, emotion, cognition, motivation; effects of pain on outcome measures of verbal scales
Individual differences	Individual traits, clinical depression, anxiety disorders, comorbid illness conditions
Social context of pain	Family, workplace, every-day life, culture
Therapeutic approaches to pain	Operant learning, associative learning, cognitive behavior therapy (CBT), acceptance and commitment (ACT)
Action—initiation of an agentic approach to pain, agency–*Proactive*	Newer direction of wellness programs—mindfulness, yoga. Emerging new conceptual models: interoception, predictive processes, Baeysian models, unstructured spontaneous cognition

Even though our work may touch on various areas of pain noted in Table 4, it is clearly situated among clinical interventions and the emerging proactive approaches to chronic pain. The biomedical model of pain with its focus on treating the structural pathology of pain as nociception has few connections to behavioral approaches to chronic pain. To the wounded soldiers of World War II we owe the beginning scientific study of pain and to Beecher (1959) the first descriptions of phantom limb pain and chronic pain conditions that were not amenable to traditional medical treatment; wounds had healed but the pain had not, opening the door to behavioral and social approaches to pain, the formulation of the biopsychosocial model of chronic pain by Melzack and Wall (1965, p. 971–978) and Melzack (2001, p. 1378–1382) and the pain avoidance operant approach of Fordyce et al. (1973). These laid the foundation for the huge expansion of the biobehavioral study of chronic pain and the treatment approaches that remain dominant today.

The most frequent description of pain is reactivity to pain and its forceful imprints on basic psychological functions of learning, emotion, cognition, and motivation that extend to social relationships, work roles, and an altered life course. Our intervention has not focused on those pain imprints. Our guides were instances of adaptive survival that navigated the impact of extreme conditions. Thus, our strategy was not one of ameliorating symptomatic impact of pain but one that simulated adaptive strategies.

Table 4 identifies the contrasting approaches and contexts. Fordyce’s pioneering work introduced the possibility that pain could be treated with behavioral approaches. His operant learning approach to pain avoidance quickly became the leading therapeutic approach in the fear avoidance model of Vlaeyen and Linton (2000, p. 317–332). Fear avoidance as the major mediator of long-term maintenance of chronic pain, became the target of treatment and prevention. The “Next Generation” of the fear avoidance model proposed a motivational approach to managing chronic pain (Crombez et al., 2012, p. 475–483). Contemporaneous work by Eccleston and colleagues focused on the disruption produced by attention demands of pain (Eccleston and Crombez, 1999, p. 356–366; Moore et al., 2012, p. 565–586), anddistraction from pain became a possible treatment strategy (Van Damme et al., 2010; Verhoeven et al., 2010; Van Ryckeghem et al., 2018).

A more recent therapeutic direction proposed motivation as a more effective way out of pain (see recent volume, Karoly and Crombez, 2018) where pain could serve as a cue for goal-directed behavior (Karsdorp et al., 2016, p. 499–507). Operant and classical conditioning approaches have remained relevant for suitable limited special cases (Madden et al., 2016), as demonstrated in the work of Flor (2017, p. S92–S96) and Flor and Turk (2011). Over the years, a number of pain therapeutic approaches were imported from mental health therapies, among them Cognitive Behavioral Therapy (CBT) for addressing cognitive distortions in appraisal or expectancy (Ehde et al., 2014). The newer Acceptance and Commitment Therapy (ACT) is gaining ascendancy (McCracken and Vowles, 2014, p. 178–87) in its emphasis on increasing valued actions in the face of pain (Hughes et al., 2017, p. 552–568). Mindfulness and other wellness approaches are increasingly supplementing traditional therapies.

The above summary of chronic pain therapies provides the clinical context as a contrast for the intervention presented in this study. The treatment of RISE does not target pain symptoms for behavioral treatment. Rather, it trains adaptations needed to prevail in the aversive unpredictable pain environment. These capacities consisted of engagement expressed in experiences of interest, curiosity, appreciation, and noticing beauty; of social relatedness expressed in empathy, compassion, helping, friendship, love; and of experiences that supported self-growth. RISE fits best into the most recent thinking about interoception body states (Tsakiris and Critchley, 2016; Fotopoulou and Trakiris, 2017) and predictive hypothesis testing aiming for the most adaptive responses. Here Tabor sees possibilities for a “pain unstuck” (Tabor et al., 2017, 2020). However, these new interoceptive models contain few suggestions for how to achieve the desired therapeutic change.

A second major feature of RISE is the structure of the content that does not aim for achieving goals as end states. Rather, it is a process in which capacities such as engaging an interest or empathy lack a distinct identifiable end state, but do take an identifiable form, such as a gradient, intensity, duration in time, and a direction or inclination. These enact a capacity intrinsic to homeostasis that resemble an approach or avoidance gradient, a process that extends beyond memory reconsolidation (Lane et al., 2015) in enacting a change in life direction. Relevant to our model is the evolving work on unstructured spontaneous cognition of Christoff (2012) and Stan and Christoff (2018), its relevance to mental health explored by Andrews-Hanna et al. (2020), and spontaneous mental experiences in extreme environments (Suedfeld et al., 2018, p. 1–33).

The concept of growth associated with pain is novel in the pain research literature, in contrast to concepts such as post-traumatic growth (Calhoun and Tedeschi, 2006) long established in the trauma literature. The efficacy of RISE to increase engagement, social relatedness, and self-growth in the context of pain could not be assessed directly. No suitable measures could be identified. We set out to develop a scale for the unpredictability of pain, and the growth in engagement, relatedness, and self-development in the midst of pain which is presently undergoing final analysis. The outcome measures we did use are validated and widely used multidimensional scales that assess a range of chronic pain symptoms, as well as emotional symptom scales, and well-being assessments.

The strongest findings are the moderate to large decreases in pain ratings that included three measures: pain intensity of WHYMPI and total pain from two comprehensive scales of POQ and SF-36. The single strongest finding is the Total POQ covering total scores from six subscales of the multidimensional POQ scale. Of note is the wide range of improvements in pain related functions: mobility, activities of daily living, vitality, negative affect, and fear, demonstrating the wide-spread effect of our self-regulation treatment strategy. For the POQ Total the intervention group decreased its pain rating by nearly one standard deviation while the control group showed no significant change, suggesting significant and large effect size improvements in how pain was experienced, and the impact it had on Veterans’ daily lives. Subjectively, this is reflected in patients’ volunteered reports: feeling no pain when one of them painted, not knowing what he might paint; spending time at woodworking, not knowing how to form the next step; playing with his grandchildren, not knowing what they would invent next. All were engaged with mobility and energy in an open embrace of the present moment.

During the past decade, pain research has increasingly recognized motivational approaches to chronic pain. Goal-directed task relevant approaches have demonstrated decreases in chronic pain (Varhoeven et al., 2011, p. 86–873; Crombez et al., 2012, p. 475–483; Romero et al., 2013, p. 135–140; Karsdorp et al., 2014, p. 92–100; Gatzounis et al., 2018, p. 109–119). We stayed with the survivor strategies of intrinsic, gradient-focused undefined end-state approaches because these are particularly conducive to exploration, self-development, self-growth, and *eudaimonia*. This self-growth emphasis is fundamentally suitable for the treatment of chronic pain where pain so often is accompanied by major declines in personal capabilities, in work roles, and social roles. An emphasis on self-growth not only emphasizes pain reduction but also underscores the restoration and further development of the person in the presence of chronic pain. Recent movement in this direction of skills building and growth is evident in studies on self-efficacy and meaning in life in relation to pain (Jackson et al., 2014, p. 800–814; Dezutter et al., 2015, p. 384–396; Karasawa et al., 2019). By designing a chronic pain intervention that enhances these self-growth pathways rather than focusing solely on pain reduction, various avenues to well-being may be achieved while creating new pathways for treatment.

Another group of three measures assessed negative affect, showing moderate to large decreases. Negative affect casts a wide net that ranges from irritability to anxiety (Affective Distress, WHYMPI); feeling depressed today, anxious today, problems concentrating today, self-esteem, feeling tense (Negative Affect, POQ); and worry about re-injury and exercise safety (Pain Fear, POQ). Pain is, indeed, a homeostatic emotion, spreading its discontent across most emotions it touches. Over the past 50 years, a large literature has investigated fear of pain as a key mediator of chronic pain (Turk and Wilson, 2010, p. 88–95) and as a major target of therapeutic treatment and intervention (Vlaeyen and Linton, 2000, p. 317–332, 2012, p. 1144–1147). Investigations of the effects of emotions on pain have consistently demonstrated that negative emotions increase pain while positive emotions significantly decrease pain (Zautra et al., 2005, p. 212–220; Wiech and Tracey, 2009, p. 987–994; Finnan and Garland, 2015, p. 177–187; Davis et al., 2018; Ong et al., 2019, p. 1140–1149). Those in the intervention group demonstrated significant decreases in negative emotion supporting this approach as an effective treatment across pain perception and negative pain related affect domains.

Our findings also included a group of tests that assessed strengths and well-being. Two measures showed significant decrease in pain interference with mobility (Mobility, POQ) and interference with vitality/energy (Vitality, POQ). Intrinsic self-regulation is loosening the powerful interference that pain has on movement when performing daily tasks. Intrinsic self-regulation is also loosening the oppressive grip that pain has on vitality expressed in physical activity, on energy level, and on strength and endurance. The research literature has focused extensively on pain interference in mobility and feelings of strength and energy and their effects on pain avoidance. Pain’s limiting effects on movement and energy have pervasive effects on activity patterns of avoidance, persistence, and pacing (Kindermans et al., 2011, p. 1049–1058). In our findings, the inhibiting effects of pain on mobility decreased while energy and vitality increased for the treatment group.

Significant gains in well-being (Emotional Well-Being, SF-36) reflect the importance of intrinsic self-regulation in the experience of well-being. The Well-Being subscale assessed a range of affects experienced during the past 4 weeks. A mix of positive and negative items represented feelings of calm, happiness, nervousness, and feeling down. Self-regulation has freed feelings of well-being from the forceful hold that pain has had. Numerous studies demonstrate that pain reduction is associated with positive feeling. However, few studies have applied positive feelings as a therapeutic/intervention approach to mitigate pain (Finnan and Garland, 2015, p. 177–187; Ong et al., 2019, p. 1140–1149). Beyond decreasing negative feelings associated with pain, this program increases positive emotions of well-being.

The intervention had no effects on symptom measures of depression, anxiety, insomnia, and PTSD as well as no effects on cognitive functions. These findings are interesting in several respects. (1) Chronicity and widespread nature of pain. The study accepted a wide range of participants with self-identified chronic pain and/or chronic pain diagnoses. The study targeted only pain and not any other emotional functions. Emotional gains were obtained in the *immediate* lowering of the Affective Distress subscale of the WHYMPI scale, particularly items covering feeling depressed today, anxious today, and problems concentrating today. Veterans in both groups reported moderate baseline levels of depression, PTSD, and insomnia. Given the chronicity of pain and distinct objectives of the RISE intervention, immediate gains in emotional functions appear more achievable in comparison to the more enduring states assessed by the standardized scales of depression, anxiety, insomnia, or PTSD that may require a more extensive intervention through expanding the duration of the intervention and expanding the methods of self-growth to more broadly target cognitive and sleep regulation. (2) Medication use. Nearly all participants were on opioid medications and/or tapering programs. Opioid medications may have suppressed gains in cognitive functions. Again, of note is that the treatment group self-reported improvement in concentration at the *immediate* present time. For this improvement to be reflected in actual performance would likely require a more extended program and include a therapeutic focus on improving cognition.

In its novel conception and method, this study joins emerging new directions in pain research. We set out to identify and test adaptive qualities that allowed individuals to endure aversive unpredictable environments. We imported these survivor strategies into chronic pain, the body’s own aversive unpredictable interoceptive environment. The survivors had spontaneously discovered methods that actually represented intrinsic self-regulation, making the application of their strategies particularly suitable for pain as a homeostatic regulatory mechanism and chronic pain representing derailed homeostasis. Unlike the spontaneous discoveries made by extreme survivors, chronic pain patients have historically been treated with approaches that were extrapolated from mental health therapies for anxiety or depression with behavioral and cognitive methods that lacked theories of cognitive and emotional functions related to chronic pain. Treatments were pursued without first understanding the problem to be treated. “Psychology of pain…has sought solutions before defining the problem,” Williams (2017, p. 150) observes (). She and others increasingly comment on the need for change in pain research. “The field seems to be in stasis,” Williams (2017, p.149) concludes. Morley, Williams, and Eccleston concur, “A paradigm shift is essential. We have gone as far as we can with the old models. The next generation of studies will need to raise the bar on quality” (Morley et al., 2013, 2004). Eccleston calls for the study of the “normal psychology of pain” that seeks to understand how pain impacts normal processes such as attention, social interaction, or task performance (Eccleston, 2013, p. 422–425).

New directions are emerging from the re-awakening of behavioral homeostasis ushered in by the ground-breaking work of Damasio on the somatic marker hypothesis and homeostatic feelings (Damasio, 1994, 2010), and of Craig’s work on lamina I neurons and the clear articulation of the afferent somatosensory tract (Craig, 2015). New thinking has extended interoceptive inference–the Bayesian inference about internal bodily states–to the somatic marker hypothesis and feelings and to physiological homeostasis that is influenced by value-based decision-making (Seth, 2013, p. 565–573, Seth, 2020, p. 270–271; Gu and FitzGerald, 2020, p. 269–270). Interoceptive sensitivity studies have assessed high vs. low cardiac interoceptive sensitivity, pain threshold, and pain tolerance (Werner et al., 2009, p. 35–42). Imaging studies have located pain-related prediction error in the anterior insula (Büchel et al., 2014, p. 1223–1239; Geuter et al., 2017, p. 1–22; Schenk et al., 2017, p. 9715–9723). In 2016, the first Interoceptive Summit convened to accelerate the understanding of the role of interoception in mental health and to consider broad topics of interoceptive assessment, afferent and efferent processing and brain-body communication, psychopathology, and also to propose a general roadmap for the future (Khalsa et al., 2018, p. 501–513). In addition, a number of new concepts are extending the reach of pain research to areas of neuroscience investigations of self-determination (di Domenico and Ryan, 2017, p. 1–13) to explore concepts applied to chronic pain (Wilson et al., 2014, p. 2074–2081; Tabor and Burr, 2019, p. 54–61), and the “intrapreneurial” self-capital as a key resource for life satisfaction and flourishing (Di Fabio et al., 2017, p. 1–5).

Our RISE intervention is part of the emerging direction that explores the complexities of homeostasis in models of interoceptive, exteroceptive, and social/environment interactions with sensitivities made possible by the new concepts and perspectives in chronic pain research. Most needed are novel clinical applications and interventions implementing the new concepts for therapeutic ends. Explorations of treatments and their efficacy will continue this paradigm shift in pain research.

The strengths of this study are theoretical, methodological, and empirical as an intervention testing the proposed theory and method. This study situates pain in homeostasis as a main regulator of survival and organismic integrity. To be sure, pain communicates threat to the body to which we react. It is also a *milieu intérieur*, not in the sense of Claude Bernard’s set points but as an environment in which the organism lives, senses, and manages to be there well or not so well. The research literature has long demonstrated the deleterious effects of ambiguous unpredictable pain, and has manipulated pain experimentally with normal and clinical populations. The adaptive survivor narratives pointed the way to being well in an aversive unpredictable context with anticipatory strategies we termed RISE expressed in experiences of engagement, social relatedness, and self-growth. The intervention method was suggested by the survivor narratives which we emulated in re-experiencing episodes of engagement and relatedness from earlier times, importing them into pain, and transforming pain into an engaged resilient response. Outcome measures showed significant improvements in pain ratings, emotional functions, and well-being ratings, confirming the preliminary efficacy of this homeostatic framework and intervention method for chronic pain. Three sets of findings support the efficacy of this intervention: comprehensive decreases in pain across multiple expressions of pain; decreases in negative affect associated with pain; and gains in experienced mobility, vitality, and emotional well-being.

Weaknesses of this study include several areas: the sample size is small; it lacks empirical validation by subsequent studies testing the present theoretical and methodological framework; the outcome measures used in this study were limited to available measures and could not directly assess engagement, relatedness, and self-growth with regard to pain The question of suitable outcome measures arises due to the newness of the intrinsic self-growth intervention under investigation. Although there are numerous available scales that measure pain coping (Brown and Nicassio, 1987; Tan et al., 2001; Riddle and Jensen, 2013), a number of mental health resilience scales (Block and Kremen, 1996; Connor and Davidson, 2003; Friborg et al., 2003) and one validated pain resilience scale (Slepian et al., 2016), these were not relevant to the behaviors we were training and the gains we hoped to achieve in these behaviors. Therefore, we determined that these outcome measures were not suitable for this study. Our concept of resilience is rooted in homeostasis and its bivalent homeostatic regulation that ebbs and flows with biological responses in interacting with the environment, here the intrinsic interoceptive context of pain. Much of homeostasis is unconscious. By the time concepts such as resilience or coping become approaches to deal with pain, these have already been shaped by the experience of daily life and the practices of community and culture in which pain is seen as an emergency in need of fixing. By contrast, our approach emerged from extreme environments and focused on survivor strategies such as interest, noticing beauty, or empathy and love rather than on changing the aversive pain environment. Therapeutic work with pain itself appeared only late in the course of the RISE intervention in the transformation module in which instances of interest, beauty or empathy were re-experienced in the presence of the pain environment that is experienced as more tolerable by noticing beauty or empathy.

The existing coping pain scales reflect a view of pain as requiring active “coping” and dealing with pain by controlling its symptoms through anything that assuages pain: seeking help, coping self-statements, resting, diverting attention, or alleviating catastrophizing (Tan et al., 2001). It assesses cognitive/affective positivity and behavioral perseverance. As with coping, the resilience scale does not assess the growth in the behaviors we aim to increase, namely engagement, relatedness, and self-growth and, therefore, is not directly applicable.

During the course of this study we decided to develop and validate an outcome measure that could assess the unpredictability of pain and the growth in engagement, relatedness, and self-growth in the presence of pain. These are not goal directed capacities to solve the problem of chronic pain but a process of homeostatic self-regulation and being well with engagement or relatedness while in the presence of pain. This scale was being developed and could not be used in the present intervention as it currently awaits final analytic treatment.

The need for follow-up evaluation remains a weakness and should be an important element in the evaluation of the efficacy of this model in future intervention studies. A final weakness of this study may arise from the heterogeneity of the pain conditions represented in the sample. The diversity of chronic pain conditions represented in the present study may obscure the applicability of the findings for specific pain conditions. An ideal sample may improve internal consistency, however, these are the patients who are using our Veterans Affairs pain clinic and, thus, represent ecological validity. Our patients likely resemble many patients with chronic pain presenting to pain clinics within the Veterans Health Administration.

## Conclusion

Homeostasis exists in support of the organism’s survival as the organism interacts with its environment. Homeostasis modulates the organism’s interaction in a bivalent process that sounds alarms and musters energy for a most adaptive survival response. In chronic conditions, the alarm is not turned off. Table 1 illustrates the bivalent process; the sympathetic pole sounds the alarm and inhibits the parasympathetic counterpart. This study describes a method for engaging an opponent process that modulates pain indirectly. The study method activates intrinsic resources present in the person’s biology, resources that current therapies are not sufficiently aware of or able to use. Our approach shows how existing capabilities can be harnessed for therapeutic ends to enhance adaptive functioning. In a randomized clinical trial of 8 weekly sessions, participants identified and practiced the main components of resilient intrinsic self-regulation of engagement and relatedness and applied these to transform chronic pain into self-growth and the design of a good life for themselves. Results showed moderate to large decreases in pain ratings in immediate pain intensity, in decreased total overall multidimensional pain scores, and decreased interference in mobility and energy. Moderate to large effect sizes for decrease in negative affect, including irritability, anxiety, present feelings of depression, and improved concentration, and self-esteem were also found. Significant gains in well-being indicated increased feelings of calm, happiness, and decreased self-doubt. Existing psychotherapies aim to correct deficits through conditioning approaches, cognitive behavior therapy, motivational and mindfulness approaches. The RISE approach introduces a new therapeutic option rooted in responses and abilities already present in a person’s repertoire to transform pain into self-growth and restructure memories that reach across space and time and shape a new life direction.

## Data Availability Statement

The datasets presented in this article are not readily available because this study pre-dates the Veterans Health Administration (VHA) implementation of the requirement to report de-identified data and VHA has grandfathered older studies. This study was not structured to release data (aside from aggregated data during publication) and is not approved by the Phoenix VA Health Care System Institutional Review Board (IRB) to do so. The IRB-approved study protocol states that only aggregate data will be released during publication. Questions regarding the dataset should be directed to VHAPHOFOIA@va.gov.

## Ethics Statement

This study was approved by the Phoenix VA Health Care System Institutional Review Board and the study participants provided their written informed consent to participate in the study.

## Author Contributions

MK was involved in conceptualization, data curation, investigation, methodology, project administration, supervision, and manuscript writing and editing. AM was involved in investigation, project administration, supervision, and manuscript editing and review. MR-H was involved in investigation, methodology, project administration, resources, and manuscript review and editing. JG-S was involved in data curation, formal analysis, and manuscript review and editing. LC was involved in data curation, formal analysis, and manuscript review and editing. JK was involved in investigation and manuscript review and editing. BS was involved in formal analysis and manuscript review and editing. All authors contributed to the article and approved the submitted version.

## Conflict of Interest

The authors declare that the research was conducted in the absence of any commercial or financial relationships that could be construed as a potential conflict of interest.
