# Supplementary material for: Adaptive Homeostatic Strategies of Resilient Intrinsic Self-Regulation in Extremes (RISE): A Randomized Controlled Trial of a Novel Behavioral Treatment for Chronic Pain
Source: Front Psychol. 2021 Apr 12;12:613341. doi: 10.3389/fpsyg.2021.613341 (PMC8074861; doi:10.3389/fpsyg.2021.613341)
Supplement: Supplementary file 1 [file Table_1.docx]

|  | Pretreatment | | | | Posttreatment | | | | ANOVA | | |
| --- | --- | --- | --- | --- | --- | --- | --- | --- | --- | --- | --- |
|  | Intervention (*n*=31) | | Control (*n*=27) | | Intervention (*n*=31) | | Control (*n*=27) | | *F* group | *F* time | *F* [group x time] |
| Measures | *M* | *SD* | *M* | *SD* | *M* | *SD* | *M* | *SD* |  |  |  |
| Pain Interference (WHYMPI) | 3.95 | 1.12 | 4.34 | .88 | 3.62 | 1.15 | 4.36 | 1.13 | 4.55 | 1.92 | 2.46 |
| Pain Life-Control (WHYMPI) | 3.86 | 1.13 | 3.52 | 1.35 | 4.37 | 1.04 | 3.31 | 1.42 | 6.95* | .67 | 3.62 |
| New Learning (RBANS List Learning) | 7.61 | 3.25 | 8.15 | 3.11 | 10.65 | 3.35 | 10.56 | 2.78 | .09 | 59.56* | .78 |
| Digit Span (RBANS) | 9.74 | 3.23 | 9.41 | 2.72 | 10.35 | 3.93 | 10.26 | 3.37 | .07 | 3.28 | .09 |
| Category Switching (DKEFS) | 10.03 | 3.04 | 11.63 | 3.55 | 11.03 | 3.41 | 9.78 | 3.27 | .05 | .30 | 12.00* |
| Story Memory RBANS) | 9.71 | 3.17 | 10.70 | 2.32 | 10.94 | 2.80 | 10.70 | 2.16 | .46 | 2.07 | 2.07 |
| PTSD (PCL-5) | 35.29 | 17.64 | 34.56 | 16.10 | 32.68 | 18.15 | 34.93 | 15.94 | .03 | .46 | .82 |
| Depression (PHQ-9) | 10.90 | 6.44 | 11.70 | 6.09 | 9.58 | 5.83 | 10.63 | 6.18 | .35 | 7.78* | .08 |
| Anxiety (GAD-7) | 8.29 | 5.66 | 7.59 | 5.72 | 7.32 | 5.68 | 8.48 | 5.64 | .03 | .005 | 2.84 |
| Insomnia (ISI) | 16.58 | 7.32 | 16.70 | 7.90 | 15.77 | 7.74 | 14.78 | 8.16 | .05 | 4.87 | .82 |
| Pain catastrophizing scale (PCS) | 19.58 | 10.37 | 20.78 | 10.80 | 17.90 | 10.82 | 20.56 | 10.72 | .55 | .81 | .48 |
| Physical symptoms (PHQ15) | 11.61 | 3.86 | 12.26 | 4.43 | 11.66 | 4.77 | 12.44 | 4.72 | .96 | .27 | .88 |

**p* = <.05
